# Phenotypic Differentiation Is Associated with Gender Plasticity and Its Responsive Delay to Environmental Changes in *Alternanthera philoxeroides* – Phenotypic Differentiation in Alligator Weed

**DOI:** 10.1371/journal.pone.0027238

**Published:** 2011-11-18

**Authors:** Wei Liu, Ru-Fang Deng, Wen-Ping Liu, Zhang-Ming Wang, Wan-Hui Ye, Lan-Ying Wang, Hong-Lin Cao, Hao Shen

**Affiliations:** 1 Key Laboratory of Plant Resources Conservation and Sustainable Utilization, South China Botanical Garden, Chinese Academy of Sciences, Guangzhou, Guangdong, People's Republic of China; 2 Department of Biology, Graduate University Chinese Academy of Sciences, Beijing, People's Republic of China; University of California, United States of America

## Abstract

Phenotypic plasticity is common in many taxa, and it may increase an organism's fitness in heterogeneous environments. However, in some cases, the frequency of environmental changes can be faster than the ability of the individual to produce new adaptive phenotypes. The importance of such a time delay in terms of individual fitness and species adaptability has not been well studied. Here, we studied gender plasticity of *Alternanthera philoxeroides* to address this issue through a reciprocal transplant experiment. We observed that the genders of *A. philoxeroides* were plastic and reversible between monoclinous and pistillody depending on habitats, the offspring maintained the maternal genders in the first year but changed from year 2 to 5, and there was a cubic relationship between the rate of population gender changes and environmental variations. This relationship indicates that the species must overcome a threshold of environmental variations to switch its developmental path ways between the two genders. This threshold and the maternal gender stability cause a significant delay of gender changes in new environments. At the same time, they result in and maintain the two distinct habitat dependent gender phenotypes. We also observed that there was a significant and adaptive life-history differentiation between monoclinous and pistillody individuals and the gender phenotypes were developmentally linked with the life-history traits. Therefore, the gender phenotypes are adaptive. Low seed production, seed germination failure and matching phenotypes to habitats by gender plasticity indicate that the adaptive phenotypic diversity in *A. philoxeroides* may not be the result of ecological selection, but of gender plasticity. The delay of the adaptive gender phenotype realization in changing environments can maintain the differentiation between gender systems and their associated life-history traits, which may be an important component in evolution of novel traits and taxonomic diversity.

## Introduction

Phenotypic plasticity is the capacity of a single genotype to produce different phenotypes in response to varying environmental conditions [1]. The resulting phenotypic flexibility may increase the organism's fitness in heterogeneous environments [2]. As a result, plasticity affects the adaptive rate of phenotypes to new environments through developmental processes [3] and may alter the interactions between individuals and their environments in ways that influence the stability and local biodiversity of populations and communities [4]. However, the role of phenotypic plasticity in adaptive diversity has historically been a contentious issue [5]. The conventional view on adaptive diversity focuses on the role of allelic substitution or quantitative genetic variation [6]. Diversity is considered a result of natural selection on the phenotypes that are affected by the genotypes, and the process of diversity is believed to be genes ‘leading’ and phenotypes ‘following’ [5]. Some biologists doubt the importance of environmentally induced traits on diversity because it usually is not immediately obvious how they can be inherited in subsequent generations [7]. Accordingly, although growing data indicate that attributing diversity to mutation and recombination fails to fully explain evolutionary routes of change [8], the contribution of plasticity to diversity through developmental processes has received little attention.

Because phenotypic plasticity encompasses diverse adaptive and also non-adaptive responses to environmental variations, no single conceptual framework can adequately explain the diverse roles of plasticity in evolutionary changes. Many characteristics of phenotypic plasticity can uniquely contribute to adaptive evolution of populations, and plasticity phenomena can be classified by these characteristics, e.g., based on reaction norm that is the particular way that phenotypes vary with environments [9]. Different terminologies have been used to describe traits that change continuously in a range of phenotypes across environments as compared to those that are expressed in discrete phenotypes. The former has been termed continuous lability [10], while the latter, polymorphism [11]. Plastic traits also show significant differences in their stabilities in varying environments. In an environment that is variable during the life span of an individual or genet, the frequency of the environmental changes could be more rapid than the ability of the individual to produce new phenotypes [12]. A growing mass of data indicate that phenotypic states of plastic traits, e.g., in lifelong plasticity and epigenetic variation, can have varying stabilities and show significant responsive delays to changing environments [13]–[15].

Phenotypic stability of plastic traits and the responsive lags induced by this stability can have important ecological effects on adaptive diversity. Such time lags can reduce the sensitivity of individuals to environmental changes, deprive these individuals of chances to react to environment variations in time, and therefore reduce their fitness significantly [12]. Researches on predator-prey and plant-herbivore systems indicate that the ability of plasticity to stabilize a population is strongly dependent on the lag between the induction time of plastic response and the timing of environmental changes; and as the lag time increases, the ability of plasticity to stabilize a population decreases, increasing the amplitude of population fluctuations [12], [16], [17].

Time lags of plastic responses can also affect the response models of phenotypic traits to environment. For example, reaction norms for discontinuous traits such as the number of digits on a guinea pig's foot would be logistic (S-shaped) with a very steep slope at the inflection point [18]. Continuously distributed traits, such as many physiological, morphological and life-history traits, typically show linear or curve-linear relationships between environments and their corresponding phenotypic values [19]. When phenotypic stability induces a significant time delay, the reaction norm of continuous traits may have an inflection point or an environmental threshold for the old phenotypic states to change to new ones. Furthermore, if phenotypic stability of plastic traits depends on the degree of environmental changes and can influence the reversibility of phenotypic states over time, it may result in two distinct ecological outcomes, canalization and plasticity [20].

Although time delays may have significant effects on phenotypic traits, it is unclear whether it is an important component contributing to phenotypic diversity, because the role of plasticity in diversity is controversial [6], [21], [22]. A large body of data indicates that regardless of selective context the origin of species differences under natural selection occurs through three steps [23]. First, the origin of a new direction of adaptive diversity starts with a population of responsive variants. That is, before the advent of a novel trait, there must be a population of variable individuals, differentially responsive, or capable of producing phenotypic variants under the influence of new inputs from the genome and/or the environment. Second, developmental recombination occurs in the population because of a new, or newly recurrent, input, resulting in novel variable phenotypes in the population and thus providing materials for selection. Finally, if the resultant phenotypic variations contribute to fitness of the individuals, selection occurs; and if the phenotypic variation has a genetic component, selection can lead to genetic accommodation that is adaptive evolution involving gene-frequency change [7]. Therefore, if developmental recombination occurs and genetic accommodation follows in the resultant phenotypes, the responsive variation would facilitate diversity.

In order to know the contribution of time lags of plasticity to phenotypic diversity, it is necessary to study: 1) the quantitative relationship between environmental changes and response time of plastic traits, which can provide the basis for establishing theoretical models to analyze phenotypic data and predict the outcome of ecological interactions between environments and phenotypic traits; and 2) if time delays affect only the time-dependent traits, or it also affects other time-independent plastic traits, or if it results in covariations of these two sets of traits. In this project, we used stamen pistillody of *Alternanthera philoxeroides*, a continuous plastic trait, to study these two issues. Stamen pistillody is one of the scenarios for the direct developmental evolution of unisexual flowers from hermaphroditic flowers, and it is considered the basis for the adaptive evolution of some species [23], [24]. The contribution of selection and genetic variation to the evolution of unisexual flowers is well known [25], but such a contribution of gender plasticity is poorly understood to our knowledge. Therefore, our data should provide information on this.

## Materials and Methods

### The species

**Figure 1:**
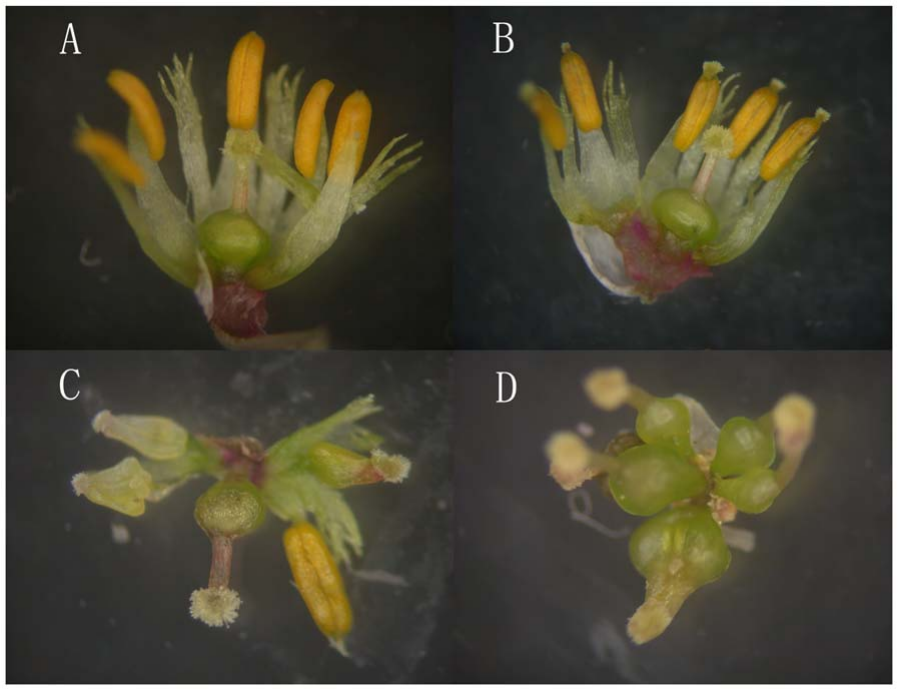
Flowers of *A. philoxeroides*. (A) a monoclinous flower, (B) a low degree gynantherous flower with only a pistillate in head of anther, (C) medium degree gynantherous flower with four incomplete pistillody stamens and a normal stamen and (D) a complete pistillody flower.

*A. philoxeroides*, alligator weed, is a stoloniferous and rhizomatous invasive plant species. Its origin is South America and it is currently invading many countries in the world [26]. It grows in both terrestrial and aquatic habitats. In China, it shows plastic continuous stamen pistillody characteristic in nature ([27], Fig. 1). The flowers of pistillody *A. philoxeroides* have no stamens but 6 carpels in two whorls, whereas the monoclinous flowers have five stamens and one carpel. There are also intermediate flower phenotypes between monoclinous and complete pistillody plants. These flower phenotypes grow in different habitats, and the gender status of individuals or populations can persist in stable habitat conditions in nature [27].

*A. philoxeroides* produces only a few fine seeds in China and its seeds do not germinate [26]. In the field, it reproduces by vegetative propagation with stolons and roots [28]. In terrestrial habitats with a cold winter, most of its stems and leaves are killed by frost. Its thick taproots can stand frost and new plants grow from them in the following spring. In aquatic habitats, most of its submerged parts can survive winter and re-sprout quickly in the following March–April. Our survey shows that, in the main distribution areas of *A. philoxeroides* in China, the monoclinous populations usually grow in sand, silt or rocky substrates; the pistillate ones in deep organic substrates, such as marsh; and both gender individuals and incomplete pistillody ones coexist in the same populations in habitats between those two groups of soil conditions. However, it is unknown how the gender ratios change with environmental variations quantitatively. The weed also shows plasticity in some phenotypic traits among these distinct environments. It has larger leaves, longer inter nodes, longer and thicker stems, larger stem pith cavity, and higher top-root ratio in aquatic than in terrestrial habitats [26]. Molecular marker analysis has revealed it has high genetic similarity within and among populations and habitats in China [28], and it is a heterozygous hexaploid cytotype in China, but it has a tetraploid and at least one heterozygous hexaploid cytotype in its origin, Argentina [29].

### Field survey

*A. philoxeroides* has a broad distribution in China. We surveyed 60 populations of it in three regions that cover its distributions in southern China: Zhengzhou (ZH) and Wuhan (WH) along the riparian zones of Yellow River and Yangtze River, respectively, and in South China Botanical Garden and the adjacent areas in Guangzhou (GZ). The populations were located in abandoned public fields with free access, e.g., deserted swamp, marsh and gravel dunes. In each population, the genders of at least 500 flowering plants of *A. philoxeroides* along a transect or all plants if there were less than 500 were recorded during peak flowering in 2003. Surveyed plants were at least 0.5 m apart, to avoid sampling individuals of the same genotypes. Based on the gender ratios, the populations were classified into pistillody (n = 23, at least 95% plants having complete pistillody flowers), monoclinous (n = 12, at least 95% plants having monoclinous flowers) or neutral populations with pistillody and monoclinous plants less than 95% (n = 25).

### Environmental variables

Our field observations indicate that *A. philoxeroides* has two major phenotypic gender states, monoclinous and pistillate. Gender diversity is from monomorphism to dimorphism. Populations with gender dimorphism show bimodality in gender as they are composed of two distinct sexual morphs that function primarily as either female or male parents. One of the dimorphisms is gynodioecy in which case one morph of the sexual system is hermaphroditic and the other is female. Distinct environment and bimodal responses of gender morphs to environment contribute to the differentiation and maintenance of gender systems [21]. To study the relationship between the gender expression of *A. philoxeroides* and environments in our project, we collected data for 7 microhabitat factors in May 2003 and 11 climate parameters (Table 1) following [21], [26], [30]–[32]. We obtained data for the 11 climate parameters from local weather stations. To collect data for the 7 microhabitat variables, each site was divided into 5 sections separated by at least 5 m from each other; in each section surveys were conducted along one transect that was randomly located and 20 to 40 m long depending on the habitat sizes and conditions. Total vegetation cover was measured by Point-Centered Quarter Method [33], starting at a point selected randomly. Along each transect, we placed four 1 m^2^ quadrates at 5 m intervals and recorded the distance from the sampling point to plants and the diameters of the plant canopies to measure vegetation cover; and we randomly took five soil samples, 15 cm in diameter and 30 cm in depth from the surface. The soil samples were analyzed for gravel proportion based on volume, soil texture, soil organic matter, pH and total N by alkaline potassium permanganate method.

**Table 1:** Eigenvalues of and the loadings of the environmental variables on the first three principal components (PC) from PCA for data of the 60 populations of *A. philoxeroides*.

	PC1	PC2	PC3
Eigenvalues	10.177	3.895	2.121
% Variance explained	56.539	21.64	11.784
Cumulative % explained	56.539	78.179	89.964
*Eigenvectors*			
*Temperature* (°C)			
Mean annual	**.964**	−.162	−.205
Mean diumal range	**−.963**	.264	−.037
Seasonality, CV	**−.958**	.145	.243
Annual range	**−.886**	.035	.460
Max. of warmest period	**.734**	−.414	.536
*Precipitation*			
Mean annual, mm	**.971**	−.220	−.073
Seasonality, CV	**−.941**	.303	−.144
Mean of wettest quarter, mm	**.958**	−.147	−.238
Mean of warmest quarter, mm	**.967**	−.173	−.182
*Radiation*			
Highest period radiation	−.111	−.359	**.925**
Lowest period radiation	**−.667**	.427	**−.609**
*Microhabitat*			
Relative water capacity	.000	.171	.152
Vegetative cover	.454	**.796**	.234
Earth pH	**−.810**	.045	−.023
Rocks and gravel content %	−.489	**−.824**	−.183
Soil content of subtract %	.488	**.829**	.174
Organic matter	.532	**.746**	.134
Total N	.567	**.717**	.132

The microhabitat variables were measured in May 2003.

**Figure 2:**
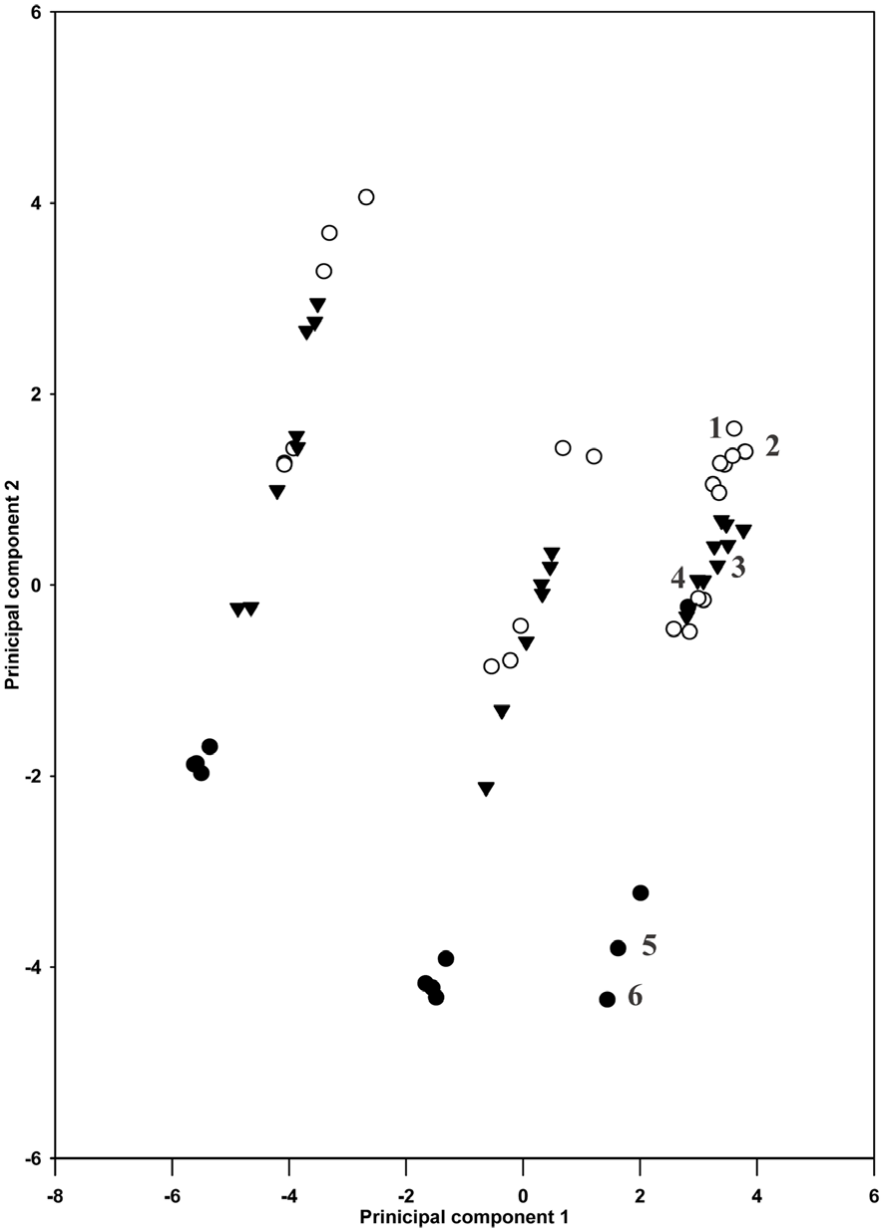
Scatterplot of various gender populations of *A. philoxeroides*. We standardized the 18 environmental variables of each population and used principal component analysis to reduce them to principal components. Pistillody (○), monoclinous (•) and neutral (▾) populations of *A. philoxeroides* were shown with respect to the first two principle components. Sites 1–6 were selected for reciprocal transplant experiments.

We standardized each of the 18 environmental variables by subtracting the mean from its values and dividing the residues by the standard deviation. Then, the transformed variables were analyzed by principal component analysis (PCA) to study how the environmental variables are associated with the gender expressions of *A. philoxeroides*. The components with eigenvalues greater than 1.0 and their un-rotated scores were used in data analysis. The first two principal components accounted for about 78% of the variation in the 18 environmental variables (Table 2). Because the pistillody and monoclinous populations are mostly separated into two clusters by PC2 (Fig. 2), PC2 represents microhabitat environmental conditions.

**Table 2:** Environmental conditions and scores on PC2 of the six sites used in the reciprocal transplant experiment.

Site	Water (%)	Vegetative cover (%)	Earth pH	RandG (%)	Soil content (%)	Organic matter (mg/g)	Total N (mg/g)	Scores onPC2
Pistillody	90	94.6	6.9	9.5	72	22	1.43	1.398
Pistillody	86	97.6	6.4	8	75	23.7	1.61	1.638
Monoclinous	50	16.1	7	73	3	0.91	0.07	−4.340
Monoclinous	40	13.9	7.1	74	2.87	0.87	0.06	−3.803
Neutral	50	91.2	6.5	11	77	15.6	0.96	0.207
Neutral	55	94.3	6.4	8	76	16.7	1.06	0.407

Water: water availability of soil, RandG: rocks and gravel content.

### Reciprocal transplant experiment

A reciprocal transplant experiment was conducted as described by Vitt [34]. Two sandy sites with monoclinous individuals, two moist sites with pistillody individuals, and two neutral sites with both pistillody and monoclinous individuals were selected based on PC2 scores from the 26 surveyed sites in Guangzhou for the experiment. The pistillody and monoclinous sites have the first and second highest and lowest scores on PC2, respectively, and the neutral sites have the average (Table 2). The pair of sites within a habitat type were uniform in climate and similar in habitat conditions. The scores (Table 2) were calculated on the basis of data of the 7 microhabitat factors collected at each of six selected sites in May 2004. They are similar to the corresponding scores based on data collected in May 2003 (data not shown, but available upon request).

In spring 2004, 50 well-developed individual *A. philoxeroides* ramets separated at least 1.5 m from each other were randomly chosen from each of the four selected pistillody and monoclinous sites, harvested and washed to remove soil. To determine the maternal effects on the offspring phenotypic traits, the gender status was recorded for each ramet. Three two-node segments with similar diameter were obtained from each ramet, randomly assigned and planted to its native site and to the sites of the other two habitats. At each experimental site, 50 pistillody and 50 monoclinous segments were planted. Plants growing from the segments were harvested in August 2004, 10 pistillody and 10 monoclinous distinct new clonally produced genets per site, for collecting data of offspring phenotypic traits (refer to details in Data collection later). Such data were not collected after 2004 because the regenerating segments forming new genets in the following years were complex and we failed to map the position of daughter ramets after 2005. Offspring gender of the subpopulations was observed in August 2004–2008.

### Date collection

All harvested plants from the experiments were divided into roots, stems, leaves, flowers and associated inflorescence parts. The lengths of branch and main stem were measured with a ruler. Then, all parts were dried in an oven at 70°C for 48 h and weighed to the nearest 0.1 mg using an electronic balance. Data for 10 phenotypic life-history traits were obtained from each genet. They belong to three groups, (1) clonal growth, (2) clonal morphology and (3) sexual reproductive traits on the basis of bimodal responses to environment for monoclinous and dimorphic populations [21], [26], [32], [34]. Group 1 includes total biomass, total branch length per genet, and main stem length. Group 2 includes branch density (number of branches/main stem length per genet), mean branch length per genet [35], ramet yield (number of ramets/genet) and biomass per ramet [21]. Group 3 contains the inflorescence traits and number of days to flowering. Because our field observations showed that not all flowers in *A. philoxeroides* inflorescences could open, to collect data for the inflorescence traits, 20 inflorescences in each site in the reciprocal experiments were randomly selected, and data were collected continuously on them once every two days in Aug. and Sept. 2004 for 30 times until wilting started. Then, the total number of opening flowers and the total number of flowers (number of opening flowers + number of bracts) per inflorescence were recorded.

In the reciprocal experiments, the number of pistillody and monoclinous flowers in each site was surveyed in May 2004, April 2005–2008. The percentages of various gender individuals were calculated for the subpopulation of each maternal gender status per site per year. Phenotypic gender of an individual was quantified by ‘femaleness’ index, *G* [36]. G is calculated using the number of floral structures and is applied to monoclinous species with a variable number of sexual structures per flower:
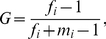
(1)where *f_i_* and *m_i_* are the sum of carpels or stamens in the flowers of individual *i*. *G* varies from 0 (monoclinous) to 1 (complete pistillody). A gender rate of a population is the mean *G* of its flowers. To simplify the surveyed results, the values of *G* were divided into ten grades, from 0 to 1.

### Measurement of population growth rate in reciprocal transplant experiment

*A. philoxeroides* genets produce ramets along their stolons. Because of the death of the aboveground parts in winter, new genets generate from the separated overwintering rootstocks of ramets in the following spring. Thus, its reproductive growth rate can be measured as the increase in the number of ramets per genet, and vegetative growth rate as the absolute and relative growth rates of biomass per genet [37], [38]. In the reciprocal transplant experiment, monoclinous individuals transplanted from their native sites to pistillody sites changed their gender into pistillody over the growing seasons from 2005 to 2008, and vice versa. Because this gender change and transplanted population growth rate were confounded, we only measured the population absolute and relative growth rate from spring to fall of 2004. From late July to early August 2004, all individuals of *A. philoxeroides* were mapped and the number of ramets was recorded. The position of daughter ramets along stolons was also mapped. Thus, in 2005 spring, we recorded the new clonal genets despite withering of the stolons and obtained ramet yield data. However, because of the gender transfer and the complex clonal configurations of the genets later, we failed to measure the clonal reproductive rate from 2005 to 2008.

### Data analysis

Statistical analyses were performed using SPSS 16.0. Data transformation was performed when necessary to meet the assumptions of homoscedasticity before analysis. The data of the 11 phenotypic traits were used in multivariate analyses. For the reciprocal transplant experiment, we analyzed the effects of maternal gender, habitat and their interactions on the offspring traits as a split-plot experimental design using analysis of covariance (ANCOVA) with the weight of transplanted segments at planting as a covariate. A one-way multivariate analysis of variance (MANOVA) was also used to analyze if the maternal gender affected the offspring traits. Regression analysis was used to study the relationship between the population gender rates and time (2004 to 2008). The slopes from this analysis were regressed with the change of environmental conditions, to study the relationship between the rate of gender change and the change of environments. The change of environments was quantified by the absolute difference in scores on PC2 between 2004 maternal and 2004 offspring sites, and we call this difference environmental stress load. All measured traits were also regressed with the absolute differences in scores on PC2.

## Results

**Figure 3:**
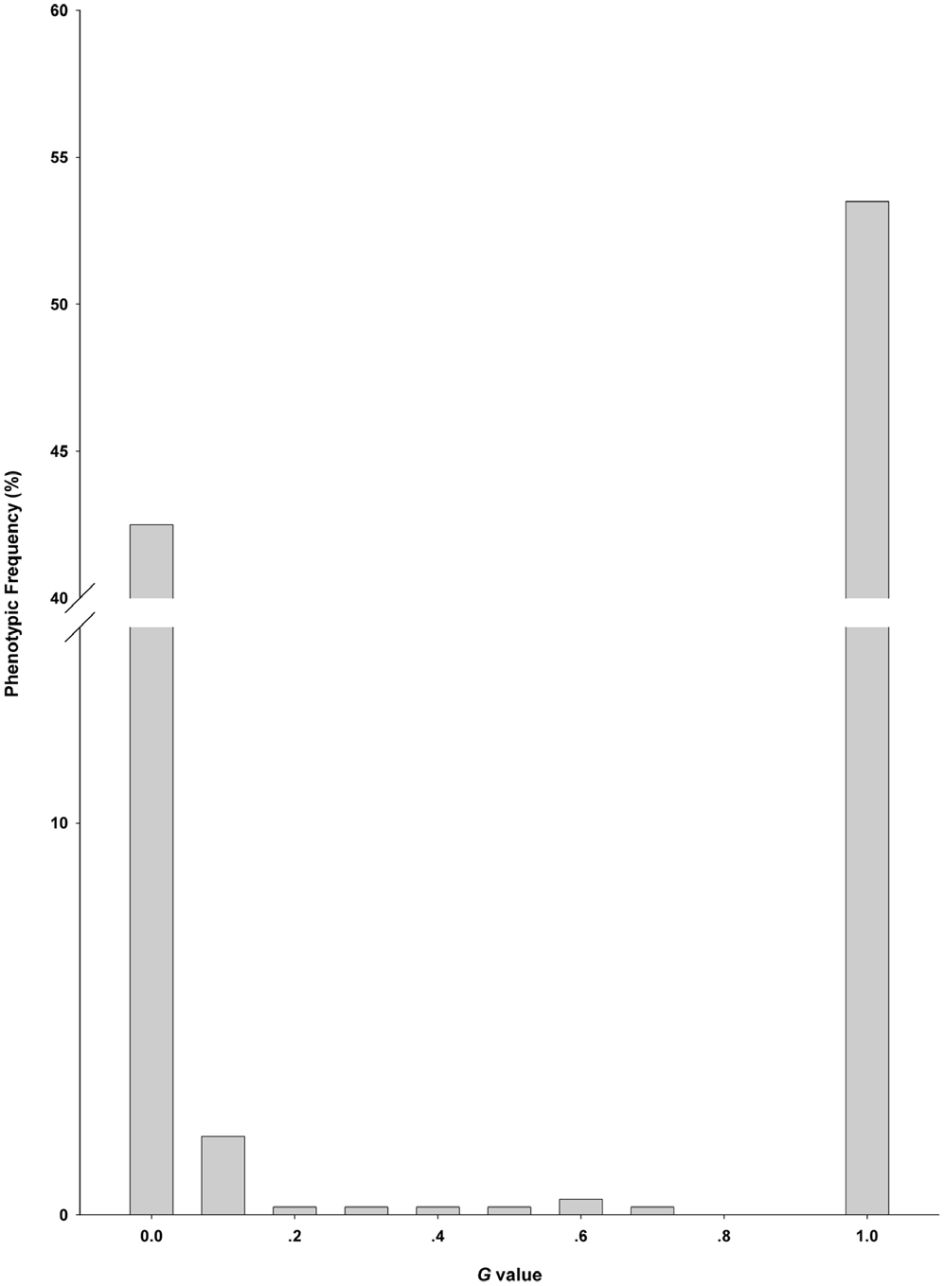
Frequency distribution of ‘femaleness’ index *G* of neutral *A. philoxeroides* populations surveyed in the field.

Our field survey indicates that *A. philoxeroides* has two major phenotypic gender states, monoclinous and pistillate. In the field, the monoclinous and pistillate genets were common, but incomplete pistillody ones were not as they were less than 2% in all our surveyed individuals (Fig. 3). In principle component analysis on the 18 environmental variables, PC2 separated monoclinous and pistillody habitats (Fig. 2) in the main distribution areas of *A. philoxeroides* in China we surveyed. Based on the absolute values of loadings of on PC2 (Table 1), microhabitat conditions (rocks and gravel content, organic matter and total N of soil, vegetative cover and subtract soil content) are most influential in determining the genders of *A. philoxeroides*.

### Response of gender plasticity to environmental variations

**Figure 4:**
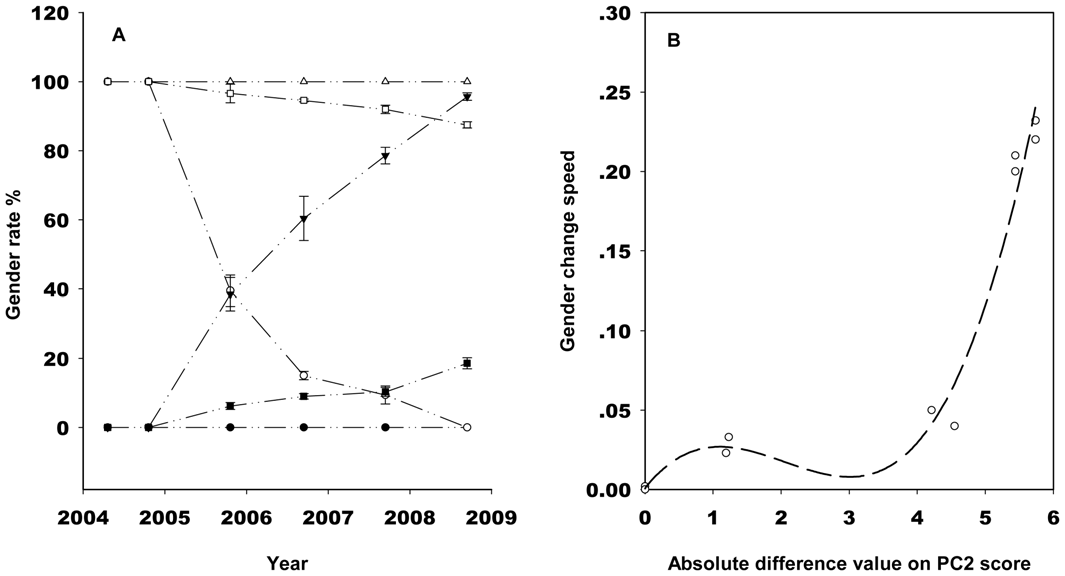
The relationship between the rate of population gender change and environmental change. (A) Gender rate (percentage of pistillody individuals) of *A. philoxeroides* over consecutive years in the reciprocal transplant populations. Monoclinous individuals planted in their own (•), in neutral (▪) (slope of regression s = 0.04, R^2^ = 0.935) and in pistillody (▴) (s = 0.232, R^2^ = 0.963) habitats over the experimental years. Pistillody individuals planted in their own (Δ), in neutral (□) (s = −0.023, R^2^ = 0.958) and in monoclinous (○) (s = −0.22, R^2^ = 0.813) habitat over the experimental years. (B) The curve-linear regression (cubic: R^2^ = 0.978, p<0.01) between cross gender transfer speed (the absolute values of the slopes of regressions from Fig. 4A) and the absolute difference values of PC2 scores between the native and the transplanted sites in reciprocal transplant experiments.

The transplanted monoclinous and pistillody ramet segments of *A. philoxeroides* maintained their maternal genders in 2004 growing season, independent of the transplanting habitats (Fig. 4A). From then on, the gender rate of the transplanted populations exhibited divergence among habitats. The pistillody and monoclinous populations maintained their gender rates over the experimental years in their native sites (Fig. 4A). When the pistillody individuals were planted in the monoclinous habitat, the proportion of monoclinous individuals in the population increased as the number of years increased, and in 2008 this proportion had approached the proportion of the monoclinous individuals planted in their native habitat, and vice versa (Fig. 4A). In the neutral habitat, the proportion of monoclinous and incomplete pistillody individuals of the maternal pistillody and that of pistillody and incomplete pistillody individuals of the maternal monoclinous increased slowly over the years (Fig. 4A). Logistic regression shows that maternal gender influences clonal offspring gender states for all sits significantly (Table 3).

**Table 3:** Results from a polytomous logistic regression model to test the effects of maternal gender, site of origin (O) and site of transplant (M and N) on the offspring gender of *A. philoxeroides*.

Parameter		β	Standard error
1a/	Maternal gender (P)	6.38^*^ c/	0.295
	Origin (O)	0b/	
	Monoclinous habitat (M)	6.361*	0.328
	Neutral habitat (N)	2.317*	0.185
	P × M	−0.838	1814.5
	P × N	−15.372	864.4
2	Maternal gender (P)	2.702*	0.671
	Origin (O)	0b/	
	Monoclinous habitat (M)	2.728*	0.843
	Neutral habitat (N)	0.862	0.651
	P × M	−14.496	3391
	P × N	−15.483	2217.5

a/ there are three gender types and the reference category is 3; 1, 2 and 3 correspond to monoclinous, incomplete and complete pistillody individuals, respectively. Pistillody individuals were used as control in this analysis.

b/ this parameter is set to zero because it is redundant.

c/ Significant at the 0.05 level.

The gender transfer speed has a significant curve linear (cubic) relationship with the absolute difference values of PC2 scores between the maternal and transplant sites (environmental stress load) in the reciprocal transplant experiments (Fig. 4B). When the environmental stress load is small from PC2 scores 0 to about 4, the gender change of the population is slow (Fig. 4B). However, when the stress load increases from PC2 scores 4 to about 6, the gender change becomes fast (Fig. 4B). Because the monoclinous and complete pistillody individuals were dominant in their own subpopulations (Fig. 3), these suggest that there is a threshold in the environmental stress load, PC2 scores 4, for the stability of the phenotypic gender states and to induce the plastic state changes of genders in *A. philoxeroides*.

### Differences in phenotypic traits between genders among habitats

**Figure 5:**
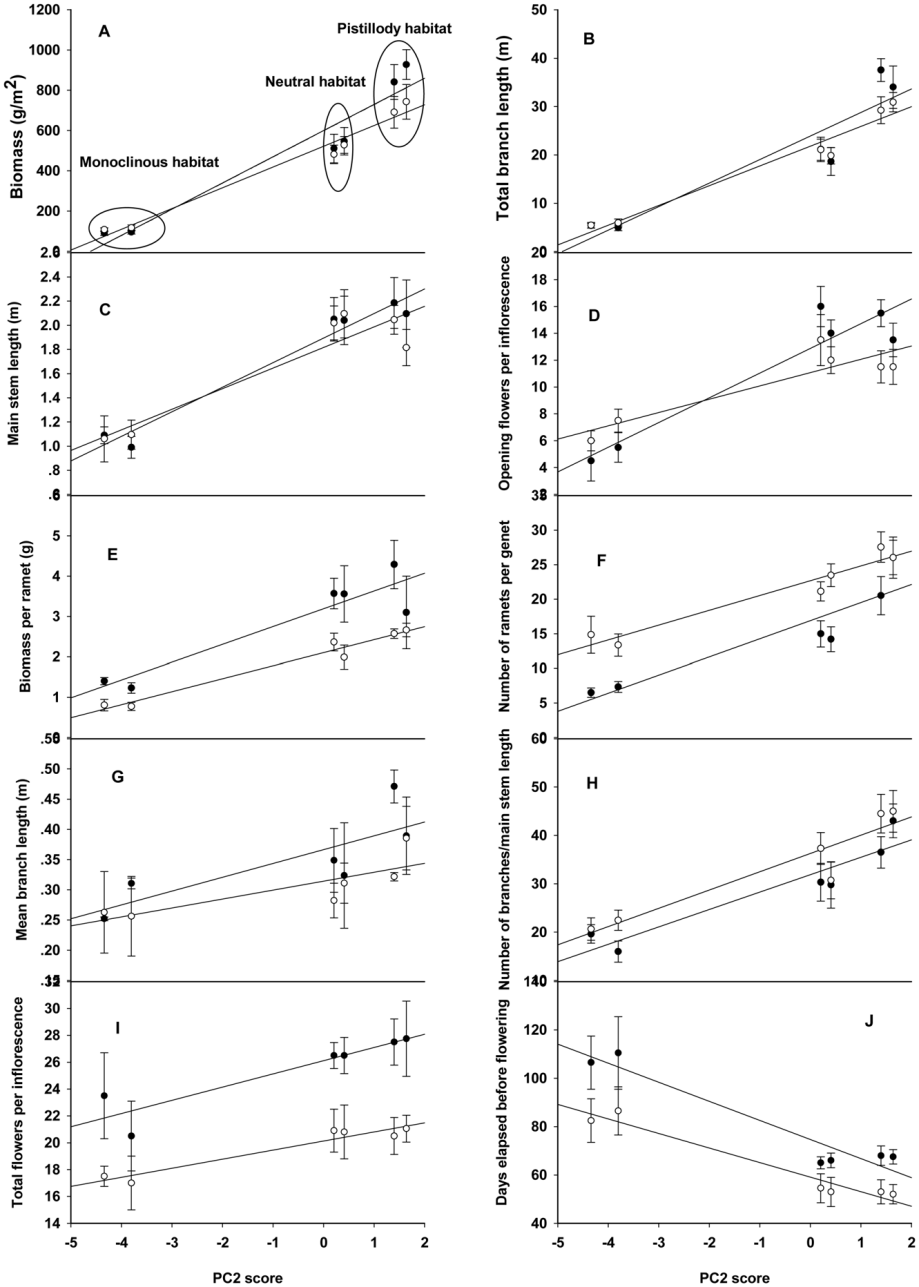
Responses of vegetative and reproductive traits of *A. philoxeroides* to environmental variations. Data were collected in Auguest 2004 for total biomass (A), total branch length per genet (B), main stem length (C), the total number of opening flowers per inflorescence (D), biomass per ramet (E), the number of ramets per genet (F), mean branch length per genet (Gg), number of branches/main stem length (H), total flowers per inflorescence (I), days elapsed before flowering (J). (•) and (○) offspring pistillody and monoclinous populations, respectively. Refer to Table 1 for PC2 scores of the 6 pistillody and monoclinous sites. The environmental variations were standardized by PC2 scores in reciprocal transplant experiments.

In the transplant experiment, values of all measured traits in August 2004, except the number of days to flowering, generally, increase with the increase of PC2 scores from monoclinous to neutral and to pistillody habitat (Fig. 5). For biomass (g/m^2^) (Fig. 5A), this positive trend is steeper for pistillody than for monoclinous populations (slope of regression for pistillody offspring is 599.6, R^2^ = 0.930, and for monoclinous offspring it is 522.1, R^2^ = 0.975). In fact, pistillody individuals have larger biomass (g/m^2^) in their native and in neutral habitats but smaller biomass (g/m^2^) in monoclinous habitat than monoclinous individuals (Fig. 5A). This is because although both pistillody and monoclinous individuals increase in size (biomass/ramet) and the number of ramets/genet with the increases in PC2 scores, the increases are faster for pistillody than for monoclinous individuals (slope of regression for number of ramets/genet of pistillody plants is 2.62, R^2^ = 0.834, and for biomass per ramet of pistillody plants it is 0.441, R^2^ = 0.859; they are 2.14, R^2^ = 0.935 and 0.322, R^2^ = 0.968 for monoclinous plants, respectively) (Fig. 5E, F).

There are similar gender-by-habitat interaction patterns as biomass (g/m^2^) for total branch length/genet, main stem length and total number of opening flowers per inflorescence measured in August 2004 (Fig. 5B–D). For these three traits, pistillody plants have larger values in pistillody habitats but smaller values in monoclinous habitat than those of monoclinous: mean values for the three traits of pistillody offspring in pistillody habitats are 35.79, 2.14 and 14.5 and monoclinous offspring in pistillody habitats are 30.07, 1.93 and 11.5; they are 5.13, 1.04 and 5 for pistillody offspring in monoclinous habitats and 5.69, 1.08 and 6.75 for monoclinous offspring in monoclinous habitats, respectively. As PC2 scores increase, mean branch length (Fig. 5G) and total number of flowers/inflorescence (Fig. 5I) increase faster for pistillody than for monoclinous plants.

### Phenotypic differences between genders

In the transplant experiment, offspring maintained their maternal genders but showed phenotypic variations between genders in other traits in 2004. Thus, the phenotypic differences between the two maternal genders are the same as those between the two offspring genders (Table 4). MANOVA detected significant differences between genders for the phenotypic traits measured (F_9, 120_ = 8.53, p<0.0001). ANCOVA results indicate that the differences between the two genders are significant for eight of the ten traits measured (Table 4). Pistillody plants have greater total number of flowers/inflorescence (FT), number of days to flowering, total branch length/genet, biomass/ramet (BR), mean branch length/genet (BL), main stem length and biomass (g/m^2^), but smaller number of branches/main stem length of a genet (branch density) and number of ramets/genet (Table 4) than monoclinous. On average, biomass/ramet of pistillody plants was 1.54 times of that of monoclinous; and a monoclinous genet produced 1.42 times as many ramets as a pistillody genet (Table 4). Branch length/genet of pistillody plants was 17% more than that of monoclinous (Fig. 5G), but branch density of monoclinous plants is 14% higher than that of pistillody (Table 4). Inflorescence of pistillody plants produced 29% more total number of flowers/inflorescence than that of monoclinous, and days to flowering is 17 days more for pistillody than for monoclinous plants (Table 4).

**Table 4:** F-values of ANCOVA results for the reciprocal transplant experiment in 2004.

ANOVA	FO	FT	Days	TBL	NB/MSL	Ramet	BR	BL	MSL	Biomass
Gender (G)	2.70	75.08*	76.21*	6.74*	5.11*	22.06*	27.87*	5.11*	0.34	5.17*
Habitat (H)	80.61*	51.30*	333.0*	688.4*	62.33*	60.07*	68.08*	7.28*	59.51*	806.2*
G×H	7.09*	2.39	6.29*	10.70*	0.05	2.08	3.49	0.45	1.23	11.43*
MSW	0.54	1.03	0.29	2.15	0.01	2.40	1.82	1.92	1.8	0
Sexual system means										
Pistillody	11.5	25.4	80.6	20.3 m	29.2/m	14.9	2.86 g	0.35 m	1.74 m	48 g
Monoclinous	10.3	19.6	63.6	18.7 m	33.4/m	21.1	1.86 g	0.3 m	1.69 m	43 g

Variables are the total number of opening flowers/inflorescence (FO), total number of flowers including opening flowers and bracts/inflorescence (FT), number of days to flowering (Days), total branch length/genet (TBL), number of branches/main stem length per genet (NB/MSL), the number of ramets/genet (Ramet), biomass/ramet (BR), mean branch length/genet (BL), main stem length (MSL) and total biomass (Biomass). Maternal transplanted segment weight (MSW) was used as covariate.

We assessed variation between sexual systems (df = 1) and among habitats (df = 2).

*: significant at 5% level.

## Discussion

We observed that after pistillody and monoclinous segments of *A. philoxeroides* were transplanted to pistillody, monoclinous and neutral habitats, their offspring maintained the maternal genders in the first year in all these habitats. From year 2 to 5, habitat dependent gender changes took place and the genders of the offspring were significantly influenced by the maternal genders. Interestingly, in these habitats from year 2 to 5, the proportions of incomplete pistillody were less than 2% (Table S1). Therefore, we consider that *A. philoxeroides* have two major plastic and reversible gender states: pistillody and monoclinous, with a delay in their responses to environmental changes.

### Cubic relationship between gender change speed and environment

Our results indicate that phenotypic gender changes of *A. philoxeroides* offspring are influenced by both environmental conditions and the maternal genders, and there is a cubic relationship between change speed of the offspring population gender rate and the environment. As incomplete pistillody is negligible, this cubic relationship may be considered a model of speed switch between the two plastic gender states across environmental conditions, and we observed that pistillody and monoclinous populations had a similar environmental response threshold of a PC2 score 4 for the switch. It seems that the threshold influences the phenotypic state stability of the plastic genders and the extent of maternal gender effects on offspring genders. It may be possible that this threshold-dependent switch controls pistillody and monoclinous developmental path ways that result in two discrete gender phenotypes in the corresponding habitats we observed.

Genetic accommodation is a mechanism of evolution wherein a novel phenotype introduced through a mutation or environmental change is molded into an adaptive phenotype through quantitative genetic changes, and it can result in environmental sensitivity changes of a plastic phenotype [39]. When a phenotype loses environmental sensitivity, it undergoes genetic assimilation, and it becomes, through evolutionary time, constitutively expressed [40]. Therefore, genetic assimilation can generate diversity by producing genetically fixed differences among populations [4]–[7]. In our study, *A. philoxeroides* produces different gender phenotypes in different habitats, and the delay of its new phenotype realization in response to habitat changes may be due to its decreased environmental sensitivity. Thus, genetic accommodation or genetic assimilation may have been occurring in *A. philoxeroides*. However, these processes have not completed since the changes of the genders in the species are reversible from pistillody to monoclinous and vice versa and therefore not genetically fixed.

### Life-history trait differentiation and gender plasticity

Fitness of an individual is measured by the number of offspring it produces in its lifetime. Because *A. philoxeroides* only produces a few seeds that do not germinate [26], the gender itself does not influence the fitness of its individuals, and such fitness must be attributed to vegetative propagation that depends on plant size [21], [32], [41]. Our results show that pistillody individuals grow larger than monoclinous in pistillody habitats, but monoclinous individuals grow larger than pistillody in monoclinous habitats in terms of biomass (g/m^2^), branch length and main stem length. Also, from monoclinous to neutral to pistillody habitats, the increases in biomass per ramet, number of ramet per genet, mean branch length, total number of opening flowers and total number of flowers per inflorescence of pistillody individuals were faster than those of the monoclinous. These indicate that pistillody individuals take advantage of productive pistillody habitats more than monoclinous ones, and monoclinous individuals tolerate poor monoclinous habitats better than pistillody ones. Therefore, the matching of the genders to habitats is likely adaptive.

One of the theories used to explain diversity in plant breeding systems is sex-allocation, and it assumes a trade-off in resource allocation between male and female functions [42]. This theory predicts that individuals should adjust their sex-allocation to their size or resource availability, and if male fitness gains decelerate faster than female fitness gains with increasing plant size and/or if self-pollination increases with plant size, then large plants should invest less in male (pollen per flower) relative to female (seeds or ovules per flower) function than small plants [43]. Our observations indicate that when monoclinous flowers change to incomplete or complete pistillody flowers in *A. philoxeroides*, one to five of the stamens in the former change to one to five carpels in the latter and pistillody individuals are larger than monoclinous ones. These would increase the female sexual reproductive potential and, at the same time, reduce or completely avoid inbreeding in pistillody individuals. The pistillody habitats are more productive and plants would grow larger in them than in poor monoclinous ones. In the pistillody habitats, strategy to reduce inbreeding should be favored, as large plant size can lead to increased selfing rates in hermaphroditic populations [21], [24]. Therefore, the differences in resource allocation to flower parts and plant size between the genders in *A. philoxeroides* we observed support the sex-allocation theory.

We also observed a trade-off between ramet number and ramet size: pistillody genets produced less number of ramets than monoclinous genets, but pistillody ramets were larger than monoclinous in biomass. This trade-off implies that pistillody and monoclinous individuals are adapted to their own habitats for the following reasons. Pistillody habitats are more productive than monoclinous, and thus competition for light and other resources among and within species may be higher in the former than in the latter. Therefore, in the former a big size individual may have competitive advantages over a small [21], [32], [41]. However, in poor monoclinous habitats such competition may be less than in pistillody habitats, and therefore allocating more resource to clonal production in ramet number than ramet size may have advantages in terms of survival and occupying new areas.

Our data show that pistillody plants produced 29% more total number of flowers per inflorescence than monoclinous plants, and they started flowering 17 days later than monoclinous plants. This could be considered a trade-off between the number of flowers and flowering time length. In general, later flowering is associated with continued vegetative growth, resulting in increased plant size and greater competitive ability [21], [32], [41], which would contribute to the advantages of big size in the pistillody habitats as discussed above. On the other hand, smaller plant size and earlier flowering in monoclinous than in pistillody plants may increase the opportunities for the monoclinous plants to finish life-history processes in the poor monoclinous habitats [21]. Furthermore, in a poor monoclinous site, the population size may be limited, and pollination agents may be lacking. Thus, a monoclinous sexual system would have advantages over pistillody to ensure successful sexual reproduction in such a population. On the contrary, the mass flowering in pistillody populations can lead to increased pollination rate and sexual reproduction success, and the forced outcrossing could result in increased fitness due to heterozygosity.

Therefore, although genders themselves do not seem to have fitness value in *A. philoxeroides*, the matching of genders to habitats and the associated flowering characteristics seem adaptive, as these provide the potential for both gender populations to survive and establish successful sexual reproductive systems to fit distinct habitats. Furthermore, the differentiations of the other plastic life-history traits associated with genders discussed so far also show adaptive responses to habitats. Therefore, it seems these two sets of traits are developmentally linked and inseparable, and we could consider them together integrated gender phenotypes or just gender phenotypes, and thus these habitat dependent integrated gender phenotypes are adaptive in *A. philoxeroides*.

A major goal of evolutionary biology is to understand how and why living things diversify. Historically, research has concentrated on genetic and ecological causes of diversification [6], [9], and many evolutionary biologists have long believed that plasticity has no relevance for the evolutionary process other than perhaps impedes it by dampening the effects of selection [22], [34], [44]–[45]. However, in recent years, research data indicate that diversification can be fully understood only by taking into account the environmental influences on the phenotype throughout the developmental processes [9]. In *A. philoxeroides*, the adaptive phenotype diversity between sexual systems is not due to ecological selection, because its life-history differentiation is associated with gender variations and selection may not act on *A. philoxeroides* gender systems directly. Furthermore, there is no genetic differentiation among habitats in *A. philoxeroides* [30]. Therefore, these and the fact that the genders are reversible and this reversibility is associated with a threshold of environmental stress load indicate the possibility that throughout evolutionary history, *A. philoxeroides* has evolved and generated different adaptive gender phenotypes through developmental plasticity. These gender phenotypes increase the phenotypic diversity and adaptability of *A. philoxeroides*. They enable the species to occupy different habitats, which may have contributed to the successful invasiveness of the species.

Phenotypic differentiation between different gender individuals has been observed in other species [25]. For example, in *Sagittaria latifolia*, a gynodioecious plant with two sexual states (female and monoecious), female plants are larger and produce heavier corms than monoecious; monoecious populations flower earlier and produce more clonal ramets and corms than female populations; and survival is highest for each sexual system in field plots that most closely resemble the habitats in which monoecious (unshaded) and female plants (shaded) populations grow [21]. These demonstrate that different gender populations have contrasting patterns of investment in traits involved with growth and reproduction, which is attributed to selection [25]. This compares with our data that indicate the matching of gender phenotypes to habitats in *A. philoxeroides* is attributable to the habitat-dependent gender plasticity, not to selection.

Although the delay of the optimum phenotype realization in a new environment could reduce the fitness of individuals [12], the significant time delay of plastic gender response to environmental change may have contributed to the maintenance of the life-history diversity between gender systems of *A. philoxeroides*. Extreme or chronic environmental changes with deleterious but non-lethal effects are common in natural populations [46]. In *A. philoxeroides*, if habitat conditions fluctuate in both monoclinous and pistillody habitats from year to year, the delay in realization of the integrated adaptive gender phenotypes and the reversibility of the genders would ensure that there are integrated adaptive gender phenotypes in these changing habitats over time. This would increase the adaptability of the species in such habitats locally over time.

One of the important ways that development influences evolution is by producing statistical associations among different phenotypic traits that in turn affect the joint evolution of these traits [47]. Because the genders and other life-history traits of *A. philoxeroides* are developmentally linked, it seems that the species switches between pistillody and monoclinous gender developmental path ways in response to the environmental conditions and produces corresponding gender individuals, and the associated phenotypic life-histroy traits make these gender phenotypes adaptive. At the same time, the discrete gender individuals result in the differentiation of these traits and thus maintain the life-history diversity between the gender systems. Adaptive semi-stable phenotypes resulting in two discrete phenotypes have also been observed in other species [13], [14], [48], [49], and the response delay of such plastic traits may be an important component in evolution of novel traits and taxonomic diversity.

## Supporting Information

Table S1 Number of various phenotypic gender individuals in the reciprocal transplant experiment from May 2004 to August 2008.
(DOC)
