# Supplementary material for: Phenotypic Differentiation Is Associated with Gender Plasticity and Its Responsive Delay to Environmental Changes in Alternanthera philoxeroides – Phenotypic Differentiation in Alligator Weed
Source: PLoS One. 2011 Nov 18;6(11):e27238. doi: 10.1371/journal.pone.0027238 (PMC3220695; doi:10.1371/journal.pone.0027238)
Supplement: Table S1 — Number of various phenotypic gender individuals in the reciprocal transplant experiment from May 2004 to August 2008. (DOC) [file pone.0027238.s001.doc]

Table S1. Number of various phenotypic gender individuals in the reciprocal transplant experiment from May 2004 to August 2008. Results are reported for subpopulations of each maternal gender status per site. Numbers of individual showing different gender status in April and August of same year are listed in bracket.

| Gender type | 2004,5 | 2004, 8 | 2005, 4 | 2006, 4 | 2007, 4 | 2008, 4 |
| --- | --- | --- | --- | --- | --- | --- |
| Pistillody→Pistillody habitat | | | | | | |
| Pistillody | 50 | 50 | 312±8(0) | 500(0) | 500(0) | 500(0) |
| Incomplete pistillody | 0 | 0 | 0(0) | 0(0) | 0(0) | 0(0) |
| Monoclinous | 0 | 0 | 0(0) | 0(0) | 0(0) | 0(0) |
| Pistillody→Neutral habitat | | | | | | |
| Pistillody | 50 | 50 | 508±14(0) | 473±2(0) | 460±6(0) | 437.5±4.5(0) |
| Incomplete pistillody | 0 | 0 | 2.5±0.5(1.5) | 4±1(2.5) | 3.5±1.5(2) | 6.5±0.5(2) |
| Monoclinous | 0 | 0 | 15.5±1.5(0) | 23±3(0) | 36.5±4.5(0) | 56±5(0) |
| Pistillody→Monoclinous habitat | | | | | | |
| Pistillody | 50 | 41±2 | 144.5±16.5(0) | 75±6(0) | 47±13(0) | 0(0) |
| Incomplete pistillody | 0 | 0 | 5.5±0.5(5) | 4.5±0.5(4.5) | 2.5±0.5(2.5) | 0(0) |
| Monoclinous | 0 | 0 | 216±19(0) | 420.5±5.5(0) | 450.5±12.5(0) | 500(0) |
| Monoclinous→Monoclinous habitat | | | | | | |
| Pistillody | 0 | 0 | 0(0) | 0(0) | 0(0) | 0(0) |
| Incomplete pistillody | 0 | 0 | 0(0) | 0(0) | 0(0) | 0(0) |
| Monoclinous | 50 | 47±3 | 466±34(0) | 500(0) | 500(0) | 500(0) |
| Monoclinous→Neutral habitat | | | | | | |
| Pistillody | 0 | 0 | 46.5±7.5(0) | 45±4(0) | 51.5±6.5(0) | 93±8(0) |
| Incomplete pistillody | 0 | 0 | 3.5±1.5(1) | 5.5±2.5(1.5) | 4±1(1.5) | 5.5±1.5(2.5) |
| Monoclinous | 50 | 50 | 697±46(0) | 449.5±6.5(0) | 445.5±7.5(0) | 401.5±6.5(0) |
| Monoclinous→Pistillody habitat | | | | | | |
| Pistillody | 0 | 0 | 133.5±16.5(0) | 302±32(0) | 393±12(0) | 478.5±5.5(0) |
| Incomplete pistillody | 0 | 0 | 5±1(3) | 1.5±0.5(1.5) | 2±1(2) | 0.5±0.5(0.5) |
| Monoclinous | 50 | 50 | 208.5±14.5(0) | 196.5±31.5(0) | 105±13(0) | 21±6(0) |
